# Valvular heart disease in pregnancy

**DOI:** 10.5830/CVJA-2016-052

**Published:** 2016

**Authors:** John Anthony, Ayesha Osman,, Mahmoud U Sani

**Affiliations:** Division of Obstetrics and Gynaecology, Groote Schuur Hospital, University of Cape Town, Cape Town, South Africa; Division of Obstetrics and Gynaecology, Groote Schuur Hospital, University of Cape Town, Cape Town, South Africa; Department of Medicine, Bayero University Kano and Aminu Kano Teaching Hospital, Kanu, Nigeria

**Keywords:** valvular, heart disease, pregnancy

## Abstract

Valvular heart disease may be a pre-existing complication of pregnancy or it may be diagnosed for the first time during pregnancy. Accurate diagnosis, tailored therapy and an understanding of the physiology and pathophysiology of pregnancy are necessary components of management, best achieved through the use of multidisciplinary clinics. This review outlines the management of specific lesions, with particular reference to post-rheumatic valvular heart disease.

## Abstract

Heart disease is one of the most common medical disorders in pregnancy. Pregnancy is associated with significant haemodynamic changes that may aggravate valvular heart disease and increase the risk of thrombo-embolic events. Valvular heart disease accounts for approximately a quarter of the cardiac diseases complicating pregnancy and is an important cause of maternal mortality, posing many challenges in management.^1^

In developing countries, valvular disease is almost exclusively the consequence of childhood rheumatic fever, although valvular dysfunction may also develop in some patients who have a prolapse of the mitral valve leaflets (Barlow’s syndrome), or ventricular dilation due to elevated afterload or cardiomyopathy.^2^ This review will be directed to the main source of valvular disease in developing countries, which is post-rheumatic disorders.

## Epidemiology of rheumatic heart disease

Rheumatic fever and its cardiac sequelae remain prevalent in developing countries.^3^ Although the Global Burden of Disease study demonstrated an overall reduction in deaths due to rheumatic heart disease (RHD) over a 20-year period, much of the change occurred in North America and Europe.^4^ The condition remains prevalent in other parts of the world, with an estimated global incidence of 282 000 new cases per year.^5^

On a global scale, the years lived with disability due to rheumatic fever, valvular heart disease caused by rheumatic disease, and heart failure related to valvular rheumatic heart disease are less encouraging, with increased rates of heart failure evident.^6^ This epidemiology is significant because it defines a condition that is preventable within the context of socio-economic upliftment, limiting overcrowding and giving sufficient access to medical care; it is also a significant cause of premature mortality. The cited estimates of mortality reflect institutional rates due to clinical disease and take no account of the pre-clinical incidence of the disease.

It has been projected that more than 15 million people suffer from RHD worldwide, which is likely a significant underestimation, according to the increasing data on subclinical RHD.^7^,^8^ RHD accounts for a major proportion of all cardiovascular disease (CVD) in children and young adults in African countries and for 17–43% of all cardiovascular disease in sub-Saharan Africa (SSA).^9^ The disease causes 400 000 deaths annually, mainly among children and young adults living in developing countries.^10^

The recently published Global Rheumatic Heart Disease registry (REMEDY) enrolled 3 343 patients (median age 28 years, 66.2% female) presenting with RHD at 25 hospitals in 12 African countries, India and Yemen. The majority (63.9%) had moderate-to-severe multi-valvular disease complicated by congestive heart failure (33.4%), pulmonary hypertension (28.8%), atrial fibrillation (AF) (21.8%), stroke (7.1%), infective endocarditis (4%) and major bleeding (2.7%). Among 1 825 women of childbearing age (12–51 years), only 3.6% were using contraception.^11^

In general, RHD accounts for about 8% of the clinical disease documented in an urban South African black population but is the presenting cardiac disease in a far higher proportion of pregnant women accessing maternity care in an African setting.^12^ In South Africa, cardiac disease in pregnancy is the most common medical disorder leading to maternal mortality and about 26% of those deaths have been attributed to complications arising from valvular heart disease.

The physiological changes of pregnancy can precipitate symptoms of cardiac disease in women who were previously asymptomatic. The management of pregnant women with valvular heart disease combines and sometimes conflicts with obstetric management of the pregnancy. Perinatal outcome becomes an additional consideration superimposed on the need for good-quality medical care. These competing interests are best managed through collaborative, combined care in a high-risk clinic attended by both obstetricians and cardiologists.^13^

## Physiology of pregnancy and heart disease

Pregnancy results in the development of a hyperdynamic circulation. Increased circulating blood volume and increased cardiac output are necessary adaptations, allowing increased uterine and placental perfusion, combined with augmented perfusion of maternal organs, which is important in pregnancy homeostasis, especially for the kidneys and skin.^14^ The changes that take place are progressive and largely determined by placental endocrine function.

The first trimester is characterised by increased cardiac output brought about by increased heart rate and stroke volume. These changes are partly induced by the onset of an expanded intravascular volume, set against peripheral arteriolar dilatation. Human chorionic gonadotrophin, which has some thyroid stimulating factor homology and activity, may also contribute to the rise in cardiac output. This is followed by progressive volume expansion secondary to physiological hyperaldosteronism with renal sodium and water retention.^15^

In the second trimester the volume expansion continues and peripheral vasodilatation dominates the pregnancy adaptation, leading to a fall in blood pressure.^16^ Early in the third trimester, the volume expansion peaks and vascular resistance rises.

Labour is accompanied by a further increase in cardiac output, which may be catecholamine mediated as a result of painful contractions. Delivery has complex haemodynamic effects, including blood loss and autotransfusion of blood from the contracted uterus immediately after delivery. In the puerperium, the extracellular fluid retention of pregnancy dissipates, with resolving peripheral oedema, and the hyperdynamic effects of pregnancy persist for days to weeks. Further cardiovascular disturbance may arise from common morbidity such as postpartum anaemia.

Pregnancy induces a procoagulant haematological profile with accelerated rates of thrombus formation and fibrinolysis.^17^ This is necessary to secure haemostasis within the choriodecidual space of the placenta and is also one of the mechanisms by which blood loss at the point of delivery is curtailed. This adaptation will increase the risk of thrombotic events in susceptible individuals.

In summary, the cardiac consequences of pregnancy are those of increased preload, reduced afterload, and increased heart rate, stroke volume and cardiac output in a hypercoagulable circulation subject to progressive change throughout pregnancy but also confronted by acutely increased demands during labour and immediately after delivery.

## Valvular heart disease

Acute rheumatic fever is a possible complication of pregnancy but is rarely seen. Most patients present with established postrheumatic valvular disease. Valvular heart disease is present in 80% of patients with heart disease during pregnancy in developing countries, with rheumatic fever as the most common aetiology.^18^ It may present for the first time during pregnancy.

Stenotic lesions that limit the ability to increase cardiac output may not be well tolerated during pregnancy and delivery. Regurgitant lesions are generally better tolerated, especially if the underlying cardiac function is normal.^19^ Occasionally, deterioration of regurgitation or left ventricular function is seen, requiring medical treatment.

## Stenotic lesions

The mitral valve is the most commonly affected valve following the development of acute rheumatic fever. A study of routine echocardiographic screening among a population of children under the age of 17 years in Mozambique and Cambodia identified mitral valve disease in 87–98% of cases.^20^ An earlier South African study showed that overall, mitral stenosis was the single most prevalent abnormality, affecting 38% of those presenting with valvular disease, although mitral incompetence was more common in the first two decades of life.^21^ The latter study identified mitral incompetence in 30% of patients, with mixed lesions making up the balance.

In the recently published REMEDY registry, children in the first decade of life presented predominantly with pure mitral regurgitation, with mixed mitral and mixed aortic valve disease emerging as a dominant mitral valve lesion from the second decade of life. Most of the cases of mitral stenosis and mitral regurgitation among other forms of valvular disease had moderate-to-severe disease.^11^

## Mitral stenosis (MS)

Rheumatic mitral stenosis is poorly tolerated in pregnancy, and it is the leading cardiac cause of maternal mortality in the developing world.^22^ It may be an incidental finding on physical examination, with many women unaware of the condition until the haemodynamic changes of pregnancy precipitate symptoms, usually in the mid-second trimester. They develop exertional dyspnoea and postural symptoms, including orthopnoea and paroxysmal nocturnal dyspnoea. Occasionally the condition may have been misdiagnosed as bronchial asthma. The classical signs of mid-diastolic rumbling murmur at the apex may be difficult to detect in patients with pulmonary oedema and a rapid tachycardia. Radiological signs of an enlarged left atrium and ECG evidence of a bifid P wave are all useful investigations. Pregnancy may, however, result in a mitralised cardiac shadow in the absence of any valvular pathology.

Pregnancy-related tachycardia and the increased blood volume are less likely to be tolerated without an increase in pulmonary capillary pressures, with increasing degrees of mitral stenosis. The increasing systemic vascular resistance of the third trimester tends to increase left-sided filling pressures further and there is a risk of pulmonary oedema during pregnancy. This risk escalates further during labour and immediately postpartum because of an increasingly hyperdynamic circulation and the acute increase in blood volume during the third stage of labour.

Significant mitral stenosis results in left atrial dilatation and an increased risk of atrial fibrillation. As pregnancy is already a hypercoagulable state, these patients are at an increased risk of developing intracardiac thrombus, and they should be anticoagulated with therapeutic low-molecular-weight heparin (LMWH).

In a South American study of 88 women with rheumatic mitral stenosis (54 of whom had moderate-to-severe mitral stenosis), eight maternal deaths occurred as a result of heart failure.^23^ In sub-Saharan Africa, a study of 50 pregnancies in women with heart disease, most of whom had rheumatic mitral stenosis, the maternal mortality rate was high at 32%.^23^

The general principles of medical management are to control the heart rate, limit the volume expansion and prevent the development of co-morbidity due to anaemia, hyperthyroidism and sepsis. In addition to controlling heart rate, beta-blockade will preserve sinus rhythm and prolong diastolic filling of the left ventricle. Atrial fibrillation and atrial flutter should be treated promptly with rate control, and early cardioversion should be considered.^24^ All drugs should be given with caution. Afterload reduction can cause reflex tachycardia with declining diastolic filling of the left ventricle, promoting cardiac decompensation. In addition, preload reduction is associated with the risk of declining cardiac output.^25^

Patients with severe mitral stenosis (valve area < 1.0 cm^2^) have high rates of complications and are likely to decompensate. In these patients and those who remain symptomatic despite medical treatment, elective percutaneous balloon valvuloplasty should be considered if the valve is suitable for the procedure, ideally during the second trimester, before 20 weeks of gestation.^19^,^26^,^27^ Patients with moderate stenosis (valve area 1.0–1.5 cm^2^) should be monitored closely and may require intervention. Mitral regurgitation may develop following valvotomy but this is usually better tolerated than a stenotic disease.^19^

Whether or not a woman is suitable for mitral valvotomy depends on the findings of echocardiographic assessment of the mitral valve apparatus. The mobility, thickness and degree of calcification of the leaflets are assessed, as is the structure of the subvalvular apparatus. Those with calcification of the commissures or significant mitral regurgitation are generally unsuitable for the procedure.^24^ If percutaneous valvuloplasty is not available, closed commissurotomy remains an alternative. Open-heart surgery should be reserved for patients without other options, when the mother’s life is threatened.^28^

## Aortic stenosis (AS)

Aortic stenosis in pregnancy is a rare condition. It is mostly associated with congenital bicuspid aortic valve (which may be linked to aortopathy and risk of aortic dissection) and is not usually the result of rheumatic disease.^29^ A diagnosis of AS is generally made pre-pregnancy and this allows for counselling, optimisation of maternal care, and planning of antenatal care. Echocardiographic quantification of AS severity and measurement of aortic diameter should be performed before pregnancy. Exercise testing is recommended in asymptomatic patients to confirm asymptomatic status and evaluate exercise tolerance, blood pressure response, arrhythmias, and the need for interventions.^28^ Features that predict a favourable outcome during pregnancy include absent symptoms, normal ECG, normal exertional blood pressure rise, aortic valve area ≥ 1 cm^2^ and normal left ventricular function.^30^

Pregnancy is usually well tolerated in asymptomatic AS, even when severe, as long as the patient remains asymptomatic during exercise testing and has a normal blood pressure response during exercise.^31^,^32^ Pregnancy should not be discouraged in asymptomatic patients, even with severe AS, when left ventricular size and function as well as the exercise test result are normal. Cardiac deterioration due to AS may be indicated by worsening breathlessness, syncope, chest pain, deterioration in left ventricular ejection fraction, a reduction or failure to increase transvalvular gradient (it should normally increase by 20% during pregnancy), and/or ischaemic ECG changes.

Symptomatic patients with severe AS or asymptomatic patients with impaired left ventricular function or a pathological exercise test should be counselled against pregnancy, and valvuloplasty or surgery should be performed before pregnancy.^28^,^32^ Medical therapy involves the use of diuretics and cautious betablockade at a low initial dose to avoid pre-syncope, syncope and hypotension. Vasodilators should be avoided. Failure of medical therapy can be managed, if gestational age allows, by delivery of the foetus, which results in significant improvement in the maternal cardiac status.

Percutaneous valvuloplasty can be undertaken in non-calcified valves with minimal regurgitation when severe symptoms persist.^33^ Valve replacement should be reserved for life-threatening symptoms, after early delivery by cesarean procedure, if this is an option. Cesarean delivery should be considered in severe, particularly symptomatic aortic stenosis.^34^

## Regurgitant lesions

The effects of rheumatic mitral regurgitation are usually ameliorated in early pregnancy by the dominant physiological change, peripheral vasodilatation. The increased plasma volume is offset by the reduction in systemic vascular resistance and consequently, the extent of the regurgitation diminishes. The plasma volume, however, peaks in the middle of the third trimester and that, together with a rise in vascular resistance, may lead to worsening regurgitation and the onset of symptoms and signs consistent with fluid overload or pulmonary oedema. Hypertension may also precipitate similar cardiovascular symptoms at an earlier stage of plasma volume expansion.

These patients respond well to diuretic therapy and usually no further intervention is necessary to secure the successful outcome of the pregnancy. Patients with severe regurgitation require expert evaluation to assess the risks and benefits of surgical intervention and the timing in relation to pregnancy. Severely symptomatic women, those with impaired left ventricular systolic dysfunction, or women with pulmonary hypertension are at high risk of maternal and foetal complications.

An enlarged left atrium increases the risk of developing atrial fibrillation. If valve surgery is indicated in women of childbearing age who have severe mitral regurgitation, valve repair should be offered when possible, thus avoiding the risks of bioprosthetic valve degeneration and early repeat surgery or anticoagulation, thrombosis and embolism associated with a mechanical valve. A woman with symptomatic mitral valve disease who is not a candidate for repair or replacement of the valve should be advised against pregnancy.

Aortic regurgitation (AR) is less common and those of rheumatic aetiology are usually associated with some degree of mitral incompetence as well.^35^ The most frequent cause of AR in women of childbearing age is also bicuspid aortic valve. The presentation during pregnancy is similar to that of mitral regurgitation and the management follows the same principles.

Chronic, moderate or even severe aortic regurgitation is usually well tolerated if left ventricular function is preserved; nevertheless, women with severe aortic regurgitation are at a risk of developing pulmonary oedema and arrhythmias during pregnancy. Valve replacement during pregnancy for treatment of aortic regurgitation is rarely required and should be considered only in women with symptoms refractory to medical therapy.^24^

Isolated tricuspid and pulmonary valve incompetence is unlikely to be of rheumatic origin and therefore not considered further here.

## Mixed lesions

Generally, the risks of mixed valvular lesions depend upon the dominant abnormality. Left-sided cardiac valvular disease is associated with greater risk, and a dominantly stenotic lesion is more likely to develop complications than patients with predominantly incompetent valves.

## Prosthetic valves

Bioprosthetic valves are associated with minimal risks during pregnancy. Conversely, mechanical valves are associated with significant maternal and foetal complications.^36^ Mechanical prosthetic valves are exposed to two risks during pregnancy, namely the twin risks of thrombosis and sepsis.

The procoagulant profile of pregnancy increases the likelihood of thrombotic events, and the need to maintain anticoagulation while protecting the foetus from exposure to anticoagulant drugs and preventing excessive haemorrhage at the time of delivery are contradictory therapeutic aims.^37^ The use of warfarin outside of pregnancy is both simple and cheap, with monitoring of anticoagulant effects made easy by measurement of the INR. In pregnancy, warfarin crosses the placenta and leads to embryopathy, foetal anticoagulation and an increased risk of pregnancy loss in all three trimesters. The alternative treatment with heparin protects the foetus from direct harm by anticoagulating only the mother; however, unfractionated heparin is only reliably used as an intravenous infusion and the use of LMWH requires monitoring of anti-Xa activity to know that the patient is in the therapeutic range.^38^

Notably, data from non-pregnant studies are not applicable to pregnancy, in which the procoagulant profile changes all the dosing schedules if a therapeutic level of anticoagulation is to be obtained. The contradictory literature pertaining to use of the different anticoagulants in pregnancy has been carefully review by Elkayam with reference to the risks of both pregnancy and the variable probability of valve thrombosis related to the specific prosthesis and the particular valve replaced.^39^ The recommendations of these authors are contained in Table 1.

**Table 1:** Our recommended approach to anticoagulation therapy for women with MPHV during pregnancy

*Higher risk*	*Lower risk*
Old-generation MPHV in mitral position, MPHV in tricuspid position, atrial fibrillation, history of TE on heparin	New-generation MPHV in mitral position and MPHV in aortic position
Warfarin (INR 2.5–3.5) for 35 to 36 weeks followed by IV UFH (aPTT > 2.5) to parturition + ASA 81–100 mg/day	LMWH SQ Q12 h (trough anti-Xa ≥ 0.6 IU/ml, peak anti-Xa < 1.5 IU/ ml) to 35 to 36 weeks, then UFH IV (aPTT > 2.0) to parturition
OR	OR
LMWH SQ Q12 h (trough anti-Xa ≥ 0.7 IU/ml, peak anti-Xa < 1.5 IU/ ml) or UFH SQ Q12 h or IV* (mid interval aPTT > 2.5) for 12 weeks, followed by warfarin (INR: 2.5–3.5) to 35 to 36 weeks, then UFH IV (aPTT > 2.5) to parturition + ASA 81–100 mg/day.	LMWH SQ Q12 h (trough anti-Xa ≥ 0.6 IU/ml, peak anti-Xa < 1.5 IU/ ml) or UFH SQ Q12 h or IV* (mid interval aPTT > 2.0) for 12 weeks followed by warfarin (INR: 2.5–3.0) until 35 to 36 weeks, then UFH IV (aPTT > 2.0) to parturition.

*IV preferred.

aPTT = activated partial thromboplastin time; ASA = acetylsalicylic acid; INR = international normalised ratio; IV = intravenous; LMWH = lowmolecular- weight heparin; MPHV = mechanical prosthetic heart valve; Q = every; SQ = subcutaneous; TE = thromboembolism; UFH = unfractionated heparin.

Of all the anticoagulants used, warfarin is the most effective agent for preventing maternal valve thrombosis but also has the highest risk of adverse pregnancy outcome. Consequently, intensive counselling is required to explain the relative risks of different treatment regimens and the anticipated complications of each approach. Long-term heparin therapy is associated with a risk of osteoporosis and heparin-induced thrombocytopenia; these adverse effects are less frequently seen with LMWHs. Both heparin and warfarin increase the risk of retroplacental haemorrhage during pregnancy, and warfarin-exposed foetuses in the first trimester risk the development of nasal hypoplasia and epiphyseal calcification. Intravenous heparin may also be complicated by line sepsis, which becomes a greater risk with increasingly prolonged periods of intravenous drug administration.

There is therefore no uniform opinion on how best to approach anticoagulation in pregnancy. Many South African units would use unfractionated heparin before 12 weeks of gestation and after 36 weeks of pregnancy, in order to have monitored control of anticoagulation that is also rapidly reversible. Warfarin is used between these gestational ages as a compromise that allows domiciliary care with ease of administration and ready access to INR monitoring. There are other ways of approaching anticoagulation, including the use of continuous warfarin or continuous LMWH. In the latter case, access to anti-Xa assays is necessary to ensure therapeutic efficacy. The question of adjuvant therapy with aspirin has been considered and certain advocates of LMWH routinely combine aspirin with LMWH throughout pregnancy.^39^

Bioprosthetic tissue valves are significantly less thrombogenic than mechanical valves, and anticoagulation is not required, unless associated arrhythmias are present.^36^ Pregnancy may be well tolerated in the presence of a normal valve structure, normal left ventricular function and absence of other cardiac lesions. Pregnancy risks increase when the valve does not function normally. Tissue valves, however, degenerate over time. In general, mitral bioprostheses degenerate faster than aortic prostheses, and the rate of degeneration is more rapid in women under 40 years of age.^40^,^41^ Therefore, women of childbearing age with bioprosthetic valves are likely to require redo heart surgery, which is an important consideration when discussing the choice of valve implant before pregnancy.^22^

Sepsis is an ever-present risk in obstetric practice at the time of delivery, although the rate of endocarditis varies widely in the reported literature, from 0–10%.^42^ The pyrexial pregnant woman with prosthetic valves deserves careful evaluation to exclude endocarditis as a diagnosis. The development of endocarditis on mechanical prosthetic valves is commonly an indication for valve replacement. The avoidance of sepsis is a priority that requires strict protocols during and after labour. These protocols include minimising the number of vaginal examinations during labour, restricting instrumentation of the genital tract during labour and delivery, scrupulous attention to anti-sepsis during the conduct of labour, ensuring that delivery of the placenta is complete, and the use of prophylactic antibiotics.

## Complicated disease

Pulmonary hypertension, diagnosed on the basis of estimated pulmonary artery pressures, evident in increased peak regurgitant velocity across the tricuspid valve, may follow mitral or aortic valvular heart disease. The development of pulmonary hypertension in this setting does not necessarily imply a worsening prognosis. A prospective Canadian study identified rheumatic valvular disease as being the single most common cause for pulmonary hypertension, accounting for 52% of cases, but was not associated with any independent increase in risk for pregnant women with left heart obstruction.^43^ The authors of this article noted that reactive pulmonary hypertension may have a different prognosis from those with primary hypertension, although there is no clarity on this issue.

The risk of maternal morbidity and mortality (17–50%) is however, generally reported to be high in all categories of pulmonary hypertension. Mortality occurs mainly in late pregnancy and after delivery, owing to heart failure, pulmonary thrombosis and arrhythmias.^44^ Recent evidence showed better outcomes in women with mild pulmonary hypertension (systolic pulmonary arterial pressure < 50 mmHg), however, no safe cut-off value is known.^45^

There is limited literature and research into the treatment of pulmonary hypertension during pregnancy.^46^ In a recent small series, no mortality occurred when nebulised iloprost was started early during pregnancy, upgraded to intravenous iloprost in some cases, with the addition of sildenafil when clinically indicated.^47^ These medications are favoured over endothelin receptor antagonists, which are teratogenic.^34^,^47^ Of the various treatment options, the use of pulmonary vasodilator therapy with sildenafil is currently under ongoing investigation and there is insufficient experience to make any recommendations.

Heart failure complicating rheumatic valvular heart disease in pregnancy has been described in 22% of women with valvular rheumatic disease presenting for care in 12 different African countries, Yemen and India.^48^ The onset of pulmonary oedema may be related to fluid overload during pregnancy, resulting from the combined alterations in intravascular volume and peripheral resistance characteristic of normal pregnancy, but may be precipitated by the injudicious use of intravenous fluids. An increasingly hyperdynamic circulation caused by the development of anaemia, subclinical hyperthyroidism, infection or the onset of labour itself may also lead to pulmonary oedema. Hypertension, whatever the precipitating mechanism, will increase left-sided filling pressures during pregnancy, with an attendant risk of pulmonary oedema. Treatment is directed towards anticipation and prevention of precipitating causes; the treatment of the cardiac lesion itself is usually combined with diuretic therapy.

An American study found that endocarditis has a rising incidence, with rates increasing from 11 per 100 000 population to 15 cases per 100 000 population.^49^ Similar trends have been seen in the United Kingdom, and the temporal relationship of this increase to the promulgation of a revised guideline advocating more conservative use of prophylactic antibiotics for individuals having interventions associated with bacteraemia is clearly demonstrable.^50^ Cardiac valves damaged by rheumatic disease are associated with turbulent blood flow, and bacteraemia triggers infection on the valve itself.

The most frequently implicated organisms are *Staphylococcus aureus*, followed by streptococci and other gram-negative organisms. Fungi can result in infection of the valve. Obstetric practice is confronted by high rates of sepsis at the time of parturition; risk factors that identify a greater probability of infection include rupture of the membranes, prolonged labour, multiple vaginal examinations during labour, instrumentation of the genital tract, surgical delivery, co-morbidity with HIV infection, and exposure to virulent organisms, especially group A streptococcal infection. Consequently, prophylactic antibiotics should be administered routinely according to established guidelines.

## Principles of combined obstetric and cardiac management

Prior to pregnancy, the severity of the cardiac condition and the cardiovascular reserve of each patient should be assessed. All women who reach childbearing age should have a discussion with their physician about the maternal and foetal risks a pregnancy would pose. In addition, drug therapy should be reviewed with the patient, particularly when potentially teratogenic drugs such as warfarin are involved. These women should be made aware of the risks of an unplanned pregnancy and should have a safe environment in which to initiate a discussion about planning a pregnancy. Contraception should also be discussed and offered to these young women.^51^ A multidisciplinary team involving the obstetrician, cardiologist, anaesthetist, neonatologist and on occasion, a cardiothoracic surgeon, is vital to the successful management of the pregnant women with heart disease.^51^

## ESC guidelines and WHO risk stratification

The European Society of Cardiology (ESC) guidelines on the management of cardiovascular disease during pregnancy recommend that all women with maternal heart disease should have a risk assessment performed at least once prior to pregnancy and then again during pregnancy.^34^ This risk assessment should be according to the modified World Health Organisation (WHO) risk classification, which integrates all known maternal cardiovascular risk factors, illustrated in Table 2, Table 3, Table 4, Table 5.

**Table 2:** Risk classification

*Risk class*	*Risk of pregnancy by medical condition*
I	No detectable increased risk of maternal mortality and no/mild increase in morbidity.
II	Small increase risk of maternal mortality or moderate increase in morbidity.
III	Significantly increased risk of maternal mortality or severe morbidity. Expert counselling required. If pregnancy is decided upon, intensive specialist cardiac and obstetric monitoring needed throughout pregnancy, childbirth and the puerperium.
IV	Extremely high risk of maternal mortality or severe morbidity; pregnancy contra-indicated. If pregnancy occurs, termination should be discussed. If pregnancy continues, care as for class III.

**Table 3:** WHO class I

• Uncomplicated, small or mild
–– pulmonary stenosis
–– patent ductus arteriosus
–– mitral valve prolapse
• Successfully repaired simple lesions (atrial or ventricular septal defect, patent ductus arteriosus, anomalous pulmonary venous drainage).
• Atrial or ventricular ectopic beats, isolated

**Table 4:** WHO class II and III

WHO II (if otherwise well and uncomplicated)
• Unoperated atrial or ventricular septal defect
• Repaired tetralogy of Fallot
• Most arrhthmias
WHO II–III (depending on individual)
• Mild left ventricular impairment
• Hypertrophic cardiomyopathy
• Native or tissue valvular heart disease not considered WHO I or IV
• Marfan syndrom without aortic dilatation
• Aorta < 45 mm in aortic disease associated with bicuspid aortic valve
• Repaired coarctation
WHO III
• Mechanical valve
• Systemic right ventricle
• Fontan circulation
• Cyanotic heart disease (unrepaired)
• Other complex congenital heart disease
• Aortic dilatation 40–45 mm in Marfan syndrome
• Aortic dilatation 45–50 mm in aortic disease associated with bicuspid aortic valve

**Table 5:** WHO class IV

• Pulmonary arterial hypertension of any cause
• Severe systemic ventricular dysfunction (LVEF < 30%, NYHA III–IV)
• Previous peripartum cardiomyopathy with any residual impairment of left ventricular function
• Severe mitral stenosis, severe symptomatic aortic stenosis
• Marfan syndrome with aorta dilated > 45 mm
• Aortic dilation > 50 mm in aortic disease associated with bicuspid aortic valve
• Native severe coarctation

## Antenatal and obstetric care

Antenatal care plans should be dependent on the risk stratification.^34^ Women in WHO class I have a very low risk and cardiology follow up during pregnancy can be limited to one or two visits. Women in WHO class II are deemed to be low or moderate risk and follow up once during each trimester of pregnancy is recommended. Women in WHO class III are high risk with an increased risk of complications. These women should be followed up at least monthly, then increasing to twice a month during the latter stages of pregnancy. Women in WHO class IV are extremely high risk. In these women, pregnancy is considered contra-indicated but if they do fall pregnant and decline termination of pregnancy, these women should have close cardiology follow up monthly if not twice monthly. In addition, factors that may contribute to cardiac decompensation, such as anaemia, infections, arrhythmias, hypertension and hyperthyroidism should be actively sought so they may be avoided or corrected.^34^,^51^

The foetal baseline scan, 13-week nuchal translucency scan and 20-week foetal anomaly scan should all be done routinely, with an extra emphasis given to excluding cardiac disease in the foetus. Growth scans should be performed as obstetrically indicated but in addition, those women with severe cardiac disease, cyanotic congenital heart disease or on medications known to cause growth restriction should have serial growth scans to detect foetal growth restriction.

The combined cardiac and antenatal clinic visits are the time when decisions regarding timing and mode of delivery, and the analgesic and anaesthesia options available are discussed and preferences are documented. Cardiac monitoring, antibiotic prophylaxis and thromboprophylaxis will need to be individualised. The entire team, including the patient, should be involved in the decision-making process.

In general, vaginal delivery with a short second stage and good analgesia is the preferred option. Caesarean section increases the risk of haemorrhage, postpartum sepsis and thrombo-embolic disease. Blood loss should be minimised at all costs and blood should be replaced promptly. Operative delivery should be limited to those patients with obstetric indications and very specific cardiac conditions.^34^,^51^

## Specific management for specific lesions

Stenotic valve diseases carry a higher risk of maternal and foetal complications than regurgitant lesions. Patients with MS, even when asymptomatic, should be advised against pregnancy and interventions should be performed prior to pregnancy. In those patients who continue the pregnancy, there should be monthly follow up. When symptoms or pulmonary hypertension develop, activity should be restricted and beta-1 selective blockers should be commenced. Low-dose diuretics can be added if symptoms persist. Therapeutic anticoagulation is recommended in patients with atrial AF or those with documented left atrial thrombosis and should also be considered in those with a large left atrium on echocardiography.

Vaginal delivery is the preferred method of delivery in mild, moderate or severe MS with NYHA class I/II with no pulmonary hypertension. Caesarean section can be considered in those patients with moderate or severe MS, with NYHA class III/IV or who have pulmonary hypertension refractory to medical therapy.

Aortic stenosis, if asymptomatic, or mild or moderate disease in pregnancy is well tolerated. Of note though is that patients with severe AS may be asymptomatic, and echocardiography is important for this diagnosis. All symptomatic patients with severe AS or even asymptomatic patients with reduced left ventricular function should be counselled against pregnancy and in these patients, surgery should be performed first. In those patients who continue the pregnancy, regular monthly follow up with echocardiography is recommended. In those patients with worsening symptoms, diuretic therapy can be administered. A beta-blocker or calcium channel antagonist can be initiated for rate control in AF, and where both of these are contra-indicated, digoxin may be considered.

In non-severe AS, vaginal delivery is preferred so as to avoid the decrease in peripheral vascular resistance during regional anaesthesia and analgesia. In patients with severe AS, particularly those who are symptomatic, caesarean section is advocated with endotracheal intubation and general anaesthesia.

Regurgitant valve disease carries a lower risk for poor maternal and foetal outcomes than stenotic lesions. Maternal risk is dependent on the severity of the regurgitation symptoms and left ventricular function.

Those patients with severe disease and symptoms, or impaired left ventricular function should be advised to have surgery prior to pregnancy. In those who continue with the pregnancy, close follow up on a monthly basis is recommended. Medical therapy is usually sufficient to manage symptoms of fluid overload. Vaginal delivery is the preferred method of delivery, and in those patients who become symptomatic, epidural anaesthesia and a shortened second stage is advisable.

Most patients with simple congenital heart lesions (these are the vast majority attending general cardiology clinics) tolerate pregnancy well. However, for those patients with more complex lesions, or those who may be taking teratogenic drugs, the issues of contraception and planning a pregnancy should be raised as soon as the young woman reaches childbearing potential.^52^

Contraceptive options are diverse and the discussion should be tailored to the individual, taking into account her underlying medical history as well as her educational and social circumstances. The practitioner will have to balance efficacy against safety but it is reasonable that in patients with severe cardiac disease where pregnancy itself poses an unacceptably high risk for the mother, then it is probably justified in leaning towards efficacy in these patients.^52^

## Conclusion

Rheumatic disease is a common and serious complication of pregnancy in developing countries. Pregnant women suffering from the sequelae of rheumatic fever benefit from the combined expertise of specialist cardiologists, obstetricians and anaesthesiologists during the pregnancy. Undiagnosed disease may also be identified for the first time during pregnancy, and the process of delivering obstetric care provides an opportunity to secure continuity of postnatal care with an emphasis on preventive therapy and contraception.
